# Supplementary material for: Microencapsulated Tuna Oil Results in Higher Absorption of DHA in Toddlers
Source: Nutrients. 2020 Jan 18;12(1):248. doi: 10.3390/nu12010248 (PMC7019307; doi:10.3390/nu12010248)
Supplement: Supplementary file 1 [file nutrients-12-00248-s001.pdf]

Supplementary Table 1. Estimated dietary intake of DHA, EPA, total n-3, total n-6 and n-6/n-3 in different countries in toddlers and young children

Country	Age group (year)	DHA (mg)	EPA (mg)	Total n-3 (g)	Total n-6 (g)	n-6/n-3
Australia [18]	2-3	24	10	0.72	6.2	8.6*
UK [19]	1-1.5	-	-	0.70	4.0	5.7*
	1.5-3	-	-	0.80	4.7	5.8*
US [20]	1-2	20	5	0.84	6.97	8.9
	2-3	20.5	5.7	0.89	8.18	10
	3-4	20.8	6.4	0.90	9.10	10.9
	4-5	21	7.1	0.93	10.16	12
Canada [21]	1.5-2	29	41	-	-	-
	2.1-3	57	95	-	-	-
	3.1-5	60	96	-	-	-
China [22]	1-3	34	-	0.7	7	9.9
	4-5	23	-	0.9	10	6.6
Korea [23]	2-4	37	22	0.6	4	8.3
Japan [24]	1-2	-	-	1.1	5.1	4.6*
	3-5	-	-	1.4	6.7	4.7*

* These data not reported in the papers, simply calculated based on total n-3 and total n-6 data reported.

Supplementary Table 2. Bioavailability of encapsulated fish oil in human studies

Age group	Number	Duration	Aim	Dose	Bioavailability	Reference
21-64	Acute: 6, Chronic: 10	4 h<br>3 weeks	To assess the bioavailability of microencapsulated tuna oil in bread (no control group)	Acute: 80 mg n-3/single dose<br>Chronic: 60 mg n-3 /d	A low dose of omega-3 consumed as microencapsulate tuna oil was bioavailable.	[37]
> 50	99	4 weeks	To assess the bioavailability of microencapsulated fish oil vs fish oil	1500 mg EPA+DHA/ d	No significant difference	[42]
Not specified	6	24 h	To check the bioavailability of microencapsulated fish oil vs fish oil	4.5 g fish oil containing 20% EPA (900 mg EPA/ d)	Encapsulated fish oil had better bioavailability compared with fish oil	[43]
18-60	10	48 h	To assess the bioavailability of emulsified oil vs fish oil capsule	71.2 mg EPA + 44 mg DHA daily	Emulsified fish oil had better bioavailability compared with fish oil capsule	[44]
19-29	28	4 weeks	To assess the bioavailability of microencapsulated fish oil vs fish oil capsule	900 mg n-3 /d	No significant difference	[45]
20-82	6	2 weeks	To assess the bioavailability of microencapsulated tuna oil vs capsular tuna oil in juice, cereal bar and yogurt.	250 mg n-3 /d	No significant difference	[28]
30-60	14	3 weeks	To assess the bioavailability of microencapsulated fish oil vs capsular fish oil in ethyl ester form	800 mg DHA + 1088 mg EPA daily	No significant difference	[48]
20-26	25	4 weeks	To assess the bioavailability of microencapsulated fish oil vs capsular fish oil.	900 mg n-3 / d	No significant difference	[47]
